# Quantification of Pseudomonas aeruginosa biofilms using electrochemical methods

**DOI:** 10.1099/acmi.0.000906.v4

**Published:** 2025-02-14

**Authors:** Lily Riordan, Perrine Lasserre, Damion Corrigan, Katherine Duncan

**Affiliations:** 1 Strathclyde Institute of Pharmacy and Biomedical Sciences, University of Strathclyde, Glasgow, G4 0RE, UK; 2 Department of Chemistry, University of Strathclyde, Glasgow, G1 1BX, UK; 3 Biosciences Institute, Faculty of Medical Sciences, Newcastle University, Newcastle Upon Tyne, NE2 4HH, UK

**Keywords:** biofilm, biofilm quantification, *Pseudomonas aeruginosa*

## Abstract

Currently, 2.29% of deaths worldwide are caused by antimicrobial resistance (AMR), compared to 1.16% from malaria and 1.55% from human immunodeficiency virus and acquired immunodeficiency syndrome. Furthermore, deaths resulting from AMR are projected to increase to more than 10 million *per annum* by 2050. Biofilms are common in hospital settings, such as medical implants, and pose a particular problem as they have shown resistance to antibiotics up to 1000-fold higher than planktonic cells because of dormant states and reduced growth rates. This is compounded by the fact that many antibiotics target mechanisms of active metabolism and are therefore less effective. The work presented here aimed to develop a method for biofilm quantification, which could be translated into the clinical setting, as well as used in the screening of antibiofilm agents. This was carried out alongside crystal violet staining, as a published point of reference. This work builds upon work previously presented by Dunphy *et al.*, in which the authors attempted to quantify the biofilm formation of *Pseudomonas aeruginosa* strain using hyperspectral imaging. Here, using electrochemical impedance spectroscopy and square wave voltammetry, the biofilm formation of two *P. aeruginosa* strains was detected within an hour after seeding *P. aeruginosa* on the sensor. A 40% decrease in impedance modulus was shown when *P. aeruginosa* biofilm had formed, compared to the media-only control. As such, this work offers a starting point for the development of real-time biofilm sensing technologies, which can be translated into implantable materials.

## Data Summary

All data associated with this work are reported within the article. Except for data underlying Figs 2, S2 and 3, which have been shared on figshare:

Figs 2 and S2: Crystal violet and square wave voltammetry quantification of PA14 and LESB58 4 h biofilms. To access the item, go to https://doi.org/10.6084/m9.figshare.28269992.v1.

Fig. 3: Ectrochemical impedance spectroscopy quantification of PA14 and LESB58 biofilms over 4 h. To access the item, go to https://doi.org/10.6084/m9.figshare.28270184.v1.

## Introduction

Biofilms are a community of bacteria, usually mixed species [1], with increased resistance to antibiotics, antimicrobials and other biocides, often with minimum biocidal concentrations of 1000-fold higher than planktonic cells [2,5]. Biofilms have been shown to afford the bacteria environmental protection [1], such as against shear stress and decreased nutrient availability [6]. Mechanisms also include the creation of a physical barrier of extracellular polymeric substances [6,8] through the sequestration of environmental and own molecules. This includes molecules and material from the environment in which the biofilm has formed, for example within an animal host, materials such as red blood cells, platelets and fibrin [8,9], as well as the cell’s own ‘junk’ DNA [1] and polysaccharides [10]. Being in a biofilm allows bacteria to maintain a larger population number, as not all the bacteria are ‘exposed’ to the outside of the biofilm, and therefore, an antibiotic, at once. This means that bacteria within a biofilm can withstand up to 1000 times higher antibiotic concentration than those not in biofilm [11,12], and can tolerate higher concentrations of organic compounds and salts [12]. In a biofilm context, medical devices and implants, such as catheters [13,15], grafts [13,16, 17] and endoscopes [2,18], are a particular issue, as they provide a surface on which the biofilm can form [2,13]. Biofilms create an obstacle for basic quantification, due to some cells entering dormancy [19], as well as cell biomass and other debris [6]. There can also be challenges with interpreting quantification results, due to the biofilm architecture and micro-colony structure [1,20]. Due to this, there are no standardized methods for biofilm quantification [1]. There are three categories for biofilm quantification: biomass assays, which quantify the extracellular matrix (ECM), along with both living and dead cells; viability assays, which quantify the living cells only; and matrix quantification, which quantifies the components of the ECM only [21]. Assays, which capture the activity of pre-formed biofilms, are of clinical relevance, as these replicate the clinical context as treatment occurs once a biofilm has become established [1].

Crystal violet (CV) staining was first used for the staining and quantification of biofilms by Fletcher [22], and since then, it has become the ‘gold-standard’ for biofilm quantification [1,4, 9, 21, 23,31]. CV staining can capture the activity of pre-formed biofilms and is one of the most common published quantification methods [6,31]. CV stains all negatively charged surface molecules and polysaccharides [21], including anionic proteins, nucleic acids and LPS [1], and has the advantage of giving data on the total biofilm biomass but also does not discriminate between live and dead cells [1,21]. It has been demonstrated to be repeatable both within and between species [21] and can be quantified using a spectrophotometer by dissolving the CV in a solvent [21,32, 33]. Prior to this advance, quantification was achieved using laborious and inaccurate microscopy cell counts both with and without CV staining [22,34]. Despite its popular use, CV can give considerable background stain [23], though this can be overcome with washing steps [1,34]. Background staining is also less significant with greater biofilm biomass, as is often observed when quantifying *Pseudomonas aeruginosa* [1,23]. However, published methods all show variations in washing and quantification techniques [1,2, 6, 21, 32, 34,36]. These variations include using no washing steps [34] or increased washing steps [23,29], as well as different solvents used to solubilize the CV, such as ethanol [4], glacial acetic acid [30] and isopropanol [29]. Lastly, a recent review found that 75% of studies quantifying biofilms had used an endpoint colourimetric assay, such as CV, and that 81% of these had used CV [31].

Electrochemical methods to detect bacteria in real time have been gaining momentum in the last few years [37,40]. One method, which has been previously employed to monitor *P. aeruginosa* growth in real-time, is square wave voltammetry (SWV) [38]. SWV is an electrochemical quantification method, which can be carried out using small sensors (0.5 cm), with the measurements solely based on medium dispersion [38]. SWV applies a range of potential differences (V) to the system, typically liquid such as growth media, and measures the current output (A). In this way, physiochemical properties of the system in the media can be determined from the analysis of the current output at a potential difference of interest [41,42]. For example, the redox-active metabolite, pyocyanin, has oxidation peaks at −0.560, –0.311 and 0.699 V [43], also reported at −0.25 V [38] and −0.37 [44]. The intensity of the peak positively correlates to the quantity of pyocyanin present in the system [38]. Other compounds are also able to be detected by SWV; for example, lysogeny broth (LB) growth media has an oxidation peak at 0.85 V [45]. Hence, this study was carried out with measurements between −0.5 and 0.5 V. As the potential difference applied is small, it only minimally affects the conditions of the system and therefore outputs robust measurements [42]. This has allowed SWV to be employed for the detection, identification and quantification of micro-organisms growing in culture [42]. The metabolites that the bacteria produce, for example, pyocyanin, change the ionic composition of the medium, thereby changing the conductivity of the media, which is measured at the working electrode at a specific potential difference [38,42]. Using this, a user can gain information about the charged molecules in the media [38], and monitoring this allows changes to the media to be observed in real time, *in situ*, and this can be applied to bacteria growing in liquid culture [40,46]. As the measurements are based on the dispersion of metabolites within the media, SWV is only able to quantify planktonic growth in real time, and not biofilm formation.

However, another electrochemical method, which has been previously employed to monitor biofilms in real time, is electrochemical impedance spectroscopy (EIS) [11,37,40]. EIS has been found to be a rapid and inexpensive point-of-care diagnostic tool, using screen-printed electrodes for less than £2 per sensor [39], and it has even been found to outperform traditional microbiological techniques [39,40]. Like SWV, EIS is also non-destructive [11]; however, it instead measures variations close to the electrode surface; the biofilm builds up directly on the surface of the sensor [39]. For EIS, measurements are based on the electrical impedance on the surface of an electrode. In EIS, a range of frequencies is passed between two electrodes, and the impedance modulus (Ω) between the electrodes is measured [47]. Using a range of frequencies allows the user to gain information about the resistive and capacitive properties of the system studied, meaning that any build-up of cells or debris on the electrodes from a forming biofilm is measured as a decrease in impedance modulus [40]. Typically, impedance values are fit to a model, such as a Randle’s equivalent circuit [37,38, 46, 48], to extract further analytical parameters [38,39]. However, changes in raw impedance modulus values have also been employed previously to detect antibiotic resistance between two strains of *S. aureus* [39]. Furthermore, these authors employed a normalization technique for EIS, which treats each electrode sensor as a closed system as impedance is sensitive; by normalizing each well against its *t*=0, any variations between sensors are considered. Both SWV and EIS allow for real-time monitoring of bacterial growth [40]; therefore, EIS has the potential to be of greater benefit for biofilm detection than other methods [38,39]. Raw impedance modulus values have also been demonstrated to be indicative of the biofilm on the sensor [40], providing easier access for point-of-care, real-time diagnostics.

*P. aeruginosa* is infamous for its prolific ability to form biofilms in inhospitable environments [6], and its ability to develop antibiotic resistance, as mentioned previously. For example, a review looking at Nepalese clinical isolates found that 42% of *P. aeruginosa* isolates were resistant to two or more antibiotics [49], whilst another study showed that more than 55% of * P. aeruginosa* clinical isolates were resistant to 12 antibiotics [50]. A large array of genetic adaptations, including horizontal gene transfer [51], and the ability to encode a large number of virulence factors [30,51] have contributed to *P. aeruginosa* being the etiological agent of 10% of all recorded nosocomial infections in the European Union [52], as well as being the leading cause of endoscope infections [2] and death amongst cystic fibrosis (CF) patients [53]. There are two commonly used laboratory strains of *P. aeruginosa*: PAO1 and PA14 [35]. PA14, originally isolated from a burn wound patient [54], has two additional pathogenicity islands to PAO1, and increased virulence [55,56]. This work focusses on PA14, as PA14 shows more consistent biofilm formation compared to PAO1 [1]. The other *P. aeruginosa* strain used in this work is LESB58, which belongs to the LES group of *P. aeruginosa* isolates, which are the most common strains in CF patients [57]. LESB58 was also the first identified *P. aeruginosa* clinical isolate [58], isolated from a CF patient in Liverpool in 1988 [53]. LESB58 is a highly virulent strain of *P. aeruginosa*, encoding 99.2% of all known *P. aeruginosa* virulence factors [59]. One of the main reasons that *P. aeruginosa* was chosen for this study was due to its ability to produce electrochemically active metabolites, such as pyocyanin, which is produced by 90–95% of *P. aeruginosa* isolates [60]. Pyocyanin production has been shown to increase with planktonic growth of *P. aeruginosa* within a closed system [38]. Furthermore, pyocyanin is reduced at −0.35 V [61], and it is the chemical signal released during the reduction process, which is measured [38]. It was therefore hypothesized that measuring the bacterially produced pyocyanin could be an accurate method to quantify the planktonic growth of *P. aeruginosa*. Furthermore, *P. aeruginosa* attachment has been seen within 2 h and plateaued at 4 h [22]. Therefore, it was hypothesized that biofilm formation of * P. aeruginosa* would be observable within 4 h using EIS.

The work presented here aims to design a model system for the monitoring of growth and inhibition of biofilm formation and develop electrochemical methods for biofilm quantification, specifically EIS and SWV, alongside a ‘gold standard’ CV as a published point of reference. Specifically, this work was carried out using the clinically relevant pathogen, *P. aeruginosa*. Two strains were chosen due to their laboratory and clinical significance: PA14 and LESB58.

## Methods

### Bacterial growth and maintenance

*P. aeruginosa* (strains PA14 and LESB58) were cultured from glycerol stocks and streaked onto LB agar and incubated (37 °C, 18 h and static). Following growth, LB liquid media (5 ml) was inoculated with a single colony and incubated (37 °C, 18 h and 250 r.p.m.). For bioactivity and biofilm assays, *P. aeruginosa* was diluted to an OD_600_ of 1, unless stated otherwise.

### Initial biofilm quantification (cuvettes)

For the initial biofilm quantification, overnight cultures of *P. aeruginosa* (PA14) were diluted to an OD_600_ of 1 and seeded into a six-well plate (1 ml, carried out in triplicate) (Corning™) and incubated (4 h, 37 °C and static). Following this, the biofilm was dislodged by pipetting the media up and down and transferred to a 1 ml cuvette. This was then read on a spectrophotometer at 600 nm. Following this, the protocol was carried out as before; however, after the 4 h incubation, the wells were washed with PBS (Sigma). This was achieved by removing the media without dislodging the biofilm, adding 1 ml of PBS to the wells, and removed gently. A further 1 ml was added, and the biofilm was dislodged and read on the spectrophotometer as before.

### Biofilm formation and quantification (96-well plates)

Both PA14 and LESB58 were diluted to an OD_600_ of 1 from overnight cultures, added (100 µl) to a clear-walled, clear-bottomed 96-well plate (carried out in triplicate) (Thermo Scientific™) and incubated (37 °C, static) for 4 h to allow for biofilm formation with minimal media evaporation. Post-incubation, absorbance was measured at 600 nm; then, the medium was removed, and the wells were washed with 100 µl dH_2_O. After air-drying [15 min, room temperature (RT)], the wells were stained with 0.1% CV in dH_2_O (w/v) for 15 min at RT, after which the CV was removed, and two further washes with PBS were carried out, and the plates were air-dried (15 min, RT). To quantify the CV-stained biofilms, 200 µl ethanol (Fisher Scientific, HPLC-grade) (95%, in dH_2_O v/v) was added to each well to solubilize the CV and the absorbance read (570 nm). The biofilm formation was normalized using min–max normalization [62], also called feature scaling, with media-only and *P. aeruginosa*-only controls to allow the data to be compared to the electrochemical data collected afterwards.

### Electrochemical biofilm quantification methods

**Fig. 1.**
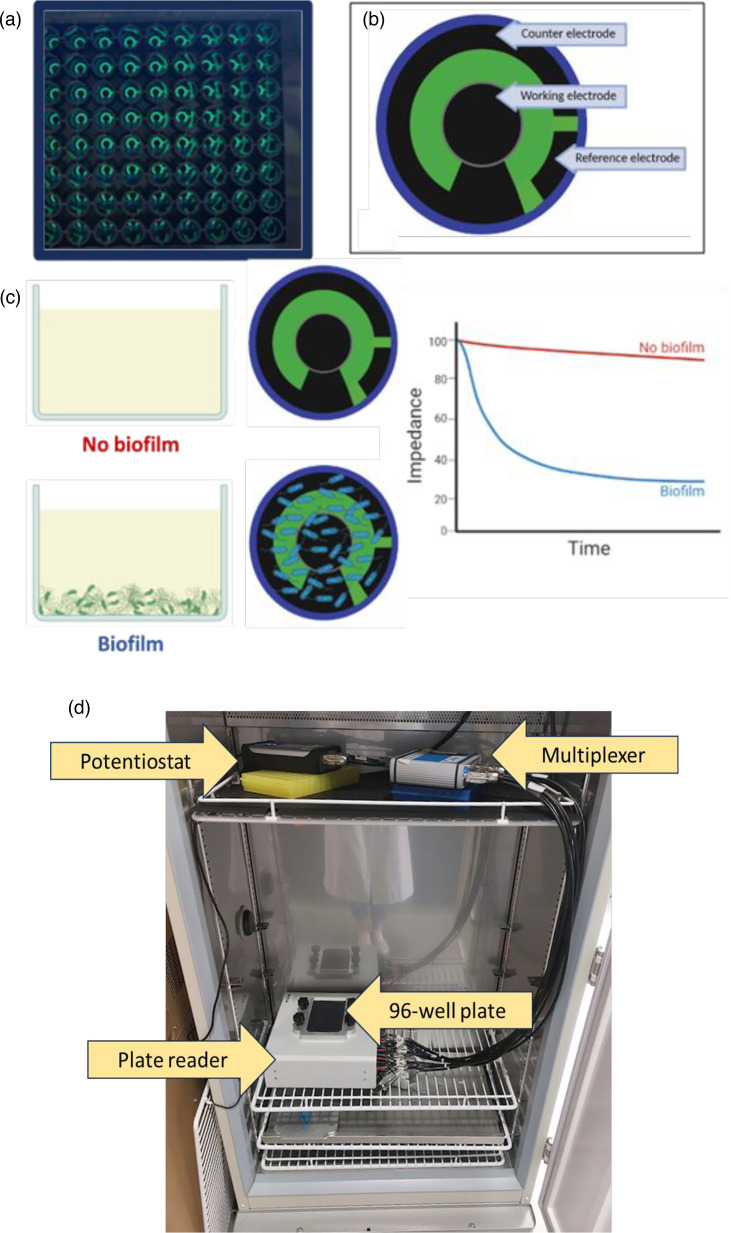
DropSens™ plate layout and biofilm formation and equipment setup. (**a**) Close-up of DropSens™ 96-well plate with carbon sensors in the base of each well. (**b**) Diagram showing the positioning of the counter, working and reference electrodes on the sensors present in the 96-well plate, (**c**) schematic of the difference observed when biofilm is present and not present on the sensor and (**d**) photo taken inside the incubator with a laptop on the bench behind the incubator (not seen). The laptop connects directly to the potentiostat, which is connected to the multiplexer on the top shelf. The multiplexer is then connected to the plate reader via 32 inputs. The multiwell plate sits on top of the plate reader; when in use, this is covered with a breathable membrane to maintain sterility. The multiplexer and potentiostat were on blocks to keep them at the same height to reduce strain on the wires.

To carry out electrochemical measurements, 96-well plates with three electrode carbon sensors in the base (Metrohm™) were used for all electrochemical measurements. These were fitted with a circuit board underneath and could be placed directly onto a specialized plate reader (DropSens Connector 96X) to input and measure electrical signals. To achieve this, desired measurements were set up as scripts on PSTrace software (version 5.9) run on a laptop (Lenovo IdeaPad 320S). A potentiostat (PalmSens4 version 1.7) and multiplexer (PalmSens MUX8-R2) were connected in sequence from the laptop to the plate reader, which enabled desired measurements to be carried out in specific wells (selected on the plate reader) in sequence, as each well forms its own circuit. The plate reader was able to operate within the incubator (Panasonic MIR-154-PE). The setup is shown in Fig. 1. Biofilms were formed in the 96-well plate with the three-electrode system in the bottom of each well at a seeding density of OD_600_ of 1, as carried out for the CV quantification and described previously. Measurements from each well were taken every 30 min for 4 h.

The laptop connects directly to the potentiostat, which is connected to the multiplexer on the top shelf. The multiplexer is then connected to the plate reader via 32 inputs. The multiwell plate sits on top of the plate reader; when in use, this is covered with a breathable membrane to maintain sterility. The multiplexer and potentiostat were on blocks to keep them at the same height to reduce strain on the wires.

SWV measurements were carried out with a 5 A current, a 3 mV step potential and a 15 Hz frequency. A range of potential differences (−0.5 to 0.5 V) was applied to the wells, and the current output (µA) was measured. This gave a peak intensity for metabolites within the media, if they are excited at a potential difference within the range. Peak height positively correlates to the quantity of the metabolite present in the media, thus allowing for quantification. The current at −0.35 V was recorded and used as the planktonic growth measurement. The data was normalized by dividing the respective well by the corresponding *t*=0 value. In this way, the variations in background noise associated with each sensor were minimized [40].

For EIS measurements, 0.1–10 000 Hz frequencies were scanned at 0.01 V AC potential (11 frequencies per decade at 67 frequencies), and the EIS spectra were measured against the open circuit potential. This output of raw impedance modulus (Ω) values was then analysed for trends, both over time at the same frequency and at a range of frequencies at the same time point. An increase in biofilm formation on the sensor correlated with a decrease in the impedance modulus at a frequency of 10 Hz. Higher frequencies contained a large amount of noise. The data was again normalized by dividing the respective well by the corresponding *t*=0 value to minimize the variations in background noise associated with each sensor [40].

### Pyocyanin concentration curve

A standard curve was required to identify SWV peak(s) of interest for pyocyanin; therefore, pyocyanin (Sigma-Aldrich) was dissolved in ethanol (100%) to a concentration of 1 mM. This stock was then diluted in dH_2_O to 100 µM and then serially diluted sevenfold in dH_2_O to 0.781 µM. These dilutions were then measured using the same SWV protocol described above, with potential differences between −0.5 and 0.5 V applied to the wells.

### Statistical analysis

For all data sets subject to statistical analysis, a Shapiro–Wilk test was performed initially to confirm that the data set was normally distributed. Following the normality test, statistical differences between samples were carried out. For the comparison of two samples, an independent sample t-test was performed. Where there were more than two samples, a one-way ANOVA was carried out to determine if there was a significant difference within the group. Following this confirmation, both Tukey’s and Dunnet’s post hoc tests were carried out, which compared all groups to each other and to the control, respectively. Significance from all tests was determined as ≤0.05, except for the Shapiro–Wilk test, in which ≤0.05 indicates that the samples are not normally distributed. All statistical analysis was carried out using SPSS [version 28.0.0.0 (190)].

## Results

First, it was important to assess *P. aeruginosa* PA14 growth under the conditions by which biofilm growth would be evaluated (37 °C, 4 h and static). This was done by dislodging the biofilm from the walls of the well (six-well plate) and then measuring the OD at 600 nm as a proxy for biofilm growth, as is standard practice for non-filamentous, non-clumping bacteria. The results showed that OD_600_ ranged from 0.060 to 0.084 with no statistical difference between replicates 1A–1C (*P*≥0.05, *n*=3) suggesting uniform and consistent measurement (Table 1) across all replicates. The final ODs of the biofilm bacteria were unexpected, as the cells were seeded at 0.2 OD_600_, and therefore, an abundance of adhered cells was anticipated. However, to more accurately use this method to quantify biofilm, it would be important to wash the planktonic cells so that only biofilm cells adhered to the plate surface would be quantified. As such, three PBS washes were introduced, and as expected, the cell density was reduced to OD_600_ 0.024, 0.031 and 0.081 (Table 1), indicating that planktonic cells had been successfully removed. There was also more variation in measurements, with the results being statistically significant from one another (*P*≤0.05), including more than a threefold difference between replicates 2A and 2C. This suggests that this method is not accurate for biofilm quantification. The main disadvantage of this method, and one that could impact the success in ‘capturing’ biofilm cells in the measurement, is that pipetting is used to transfer the culture to the cuvette for measurements in the spectrophotometer. There was no method used to determine if all the biofilm cells had been removed from the wells for quantification. This may account for the discrepancies between replicates as well as the low OD_600_ result compared to the seeding density. As such, the results were expected, and, next, the ‘gold standard’ method of biofilm quantification, CV staining, was assessed. This allowed for biofilms to be quantified within a 96-well plate (necessary for the chosen electrochemical measurements later) and therefore circumvented the cell-removal issues experienced during OD measurement.

**Table 1.** Biofilm formation of *P. aeruginosa* quantified spectroscopically. OD_600_ of *P. aeruginosa* (PA14) biofilm after 4 h incubation at 37 °C with (2A–C) (*P*≤0.05, *n*=3) and without (1A–C) (*P*≥0.05, *n*=3) PBS washing

Replicate	OD_600_ (nm)	PBS wash
1A	0.083	No
1B	0.060	No
1C	0.084	No
2A	0.081	Yes
2B	0.031	Yes
2C	0.024	Yes

Following on from the initial biofilm formations in six-well plates, decreasing seeding ODs of *P. aeruginosa* (PA14) were introduced to observe if decreasing quantities of biofilm could be detected. The lack of staining in the CV control after the biofilms were washed confirmed that both the planktonic cells and the background stain were removed. Also, there was a visual decrease in the quantity of stain for decreasing OD_600_ of *P. aeruginosa*.

**Fig. 2.**
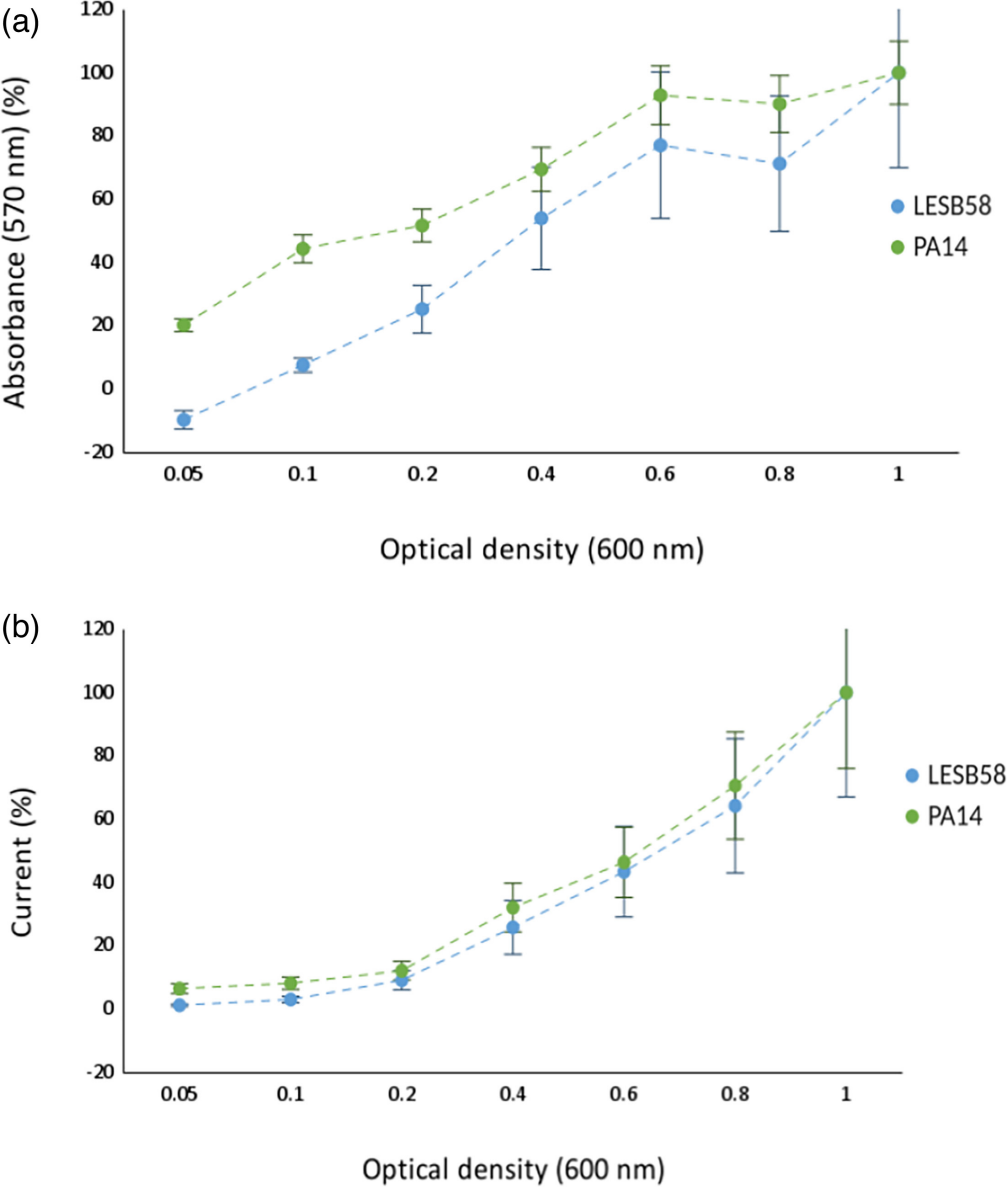
Quantification of *P. aeruginosa* [PA14 (green) and LESB58 (blue)] biofilms with increasing seeding densities (OD_600_ 0.05–1). (**a**) Measured after 4 h using spectroscopy readings at 570 nm of solubilized CV in ethanol (error bars show sd, *n*=3, *r*^2^=94 and 92%, respectively). (**b**) Current (μA) measured over 4 h at −0.35 V, normalized 4 h time point shown (error bars show sd, *n*=3 and *r*^2^=91% for both).

From this, it was necessary to quantify the CV-stained biofilm, and therefore, 95% ethanol (v/v) was used to solubilize the biofilm-bound CV. The biofilms were formed from seeding densities of 0.05–1 OD_600_. The addition of ethanol to the CV-stained wells solubilizes the CV from the walls of the wells into the solvent, allowing for spectrophotometric quantification of the CV (Fig. 2a). The CV quantification of both PA14 and LESB58 at increased seeding densities (Fig. 2a) had clear linear trend lines. As the data was normalized, the end value was 100% biofilm formation, showing a 79.2% increase in biofilm formation between the lowest and highest seeding ODs for PA14. LESB58 showed a 109.7% increase. These both positively correlated to the initial OD that the *P. aeruginosa* were seeded at *r*^2^ values of 94% and 92%, respectively, indicating the percentage of explained variation of the total variation. Furthermore, there was an overall increase in the sd at the higher starting ODs, particularly compared with those at ODs 0.05 and 0.1 (Fig. 2a). Next, it was of interest to determine if *P. aeruginosa* biofilm formation could be viewed in real-time, rather than as an endpoint, as with CV quantification.

As CV quantification provided challenges with accurate and repeatable measurements, as well as only allowing for endpoint reads, we chose to develop methods, which enabled electrochemistry to be used to monitor biofilm formation in real time and *in situ*. As mentioned previously, *P. aeruginosa* produces an electrochemically active secondary metabolite, pyocyanin, which can be measured using electrochemical techniques and used as a proxy for growth [38]. Briefly, a range of potential differences (−0.5 to 0.5 V) was applied to the wells, and the current output (µA) was measured. Peak height positively correlates to the quantity of the metabolite present in the media, thus allowing for quantification. As proof of concept, increasing concentrations of a pyocyanin standard were quantified using SWV, with a potential difference at −0.35 V, as this is the potential at which pyocyanin is reduced [61], to create a pyocyanin concentration curve (Fig. S1, available in the online Supplementary Material). This has been carried out previously on gold screen-printed electrodes [38]; however, it was important to carry out this initial concentration curve to show that this also works in this system (carbon screen-printed electrodes). This showed a strong positive correlation between the pyocyanin concentration of a solution and the resulting current (μA) (*r*^2^=99.9%). As pyocyanin production has been shown to increase with the planktonic growth of *P. aeruginosa* in a closed system [38], it was therefore hypothesized that measuring the bacterially produced pyocyanin could be an accurate method to quantify the planktonic growth of the *P. aeruginosa*.

As such, the next experiment used the same setup, but with both *P. aeruginosa* PA14 and LESB58 at increased seeding densities, as carried out earlier. The results showed a similar trend to the CV data (Fig. 2b). As hypothesized from the concentration curve, there was an increased current (μA) output at increased seeding densities (shown as a percentage as the data have been normalized). For example, for PA14, OD 0.05=6.4% increase in current, compared to OD 0.8=70.6%. Similar trends were seen for LESB58, OD 0.05=1.2% and OD 0.8=64.2%. This increased current output demonstrates increased pyocyanin production and, subsequently, the density of *P. aeruginosa* cells. As both sets of data were normalized to the current at the highest concentration of pyocyanin (OD_600_=1), Fig. 2b does not show the differences in pyocyanin production between PA14 and LESB58. Looking at the current output data prior to normalization (Fig. S2), it can be observed that LESB58 produces more pyocyanin than PA14; LESB58=12.7 μA, compared to 2.2 μA for PA14, both at OD_600_ 1. This is because LESB58 has increased virulence compared to PA14, and pyocyanin is a virulence factor of *P. aeruginosa*. The pyocyanin concentration curve had an *r*^2^ value of 99.9%, compared to 91% for the SWV data (both PA14 and LESB58), with *r*^2^ indicating the percentage of explained variation of the total variation. This is surprising, as the concentration curve in Fig. S1 is pyocyanin and media only, whereas *P. aeruginosa* cultures produce other metabolites in addition to pyocyanin. These additional metabolites, such as pyoverdine, add additional variation to these wells, which is not measured by the concentration curve. Next, it was of interest to determine if biofilm formation could be observed over the 4 h, rather than an endpoint read, as with CV.

**Fig. 3.**
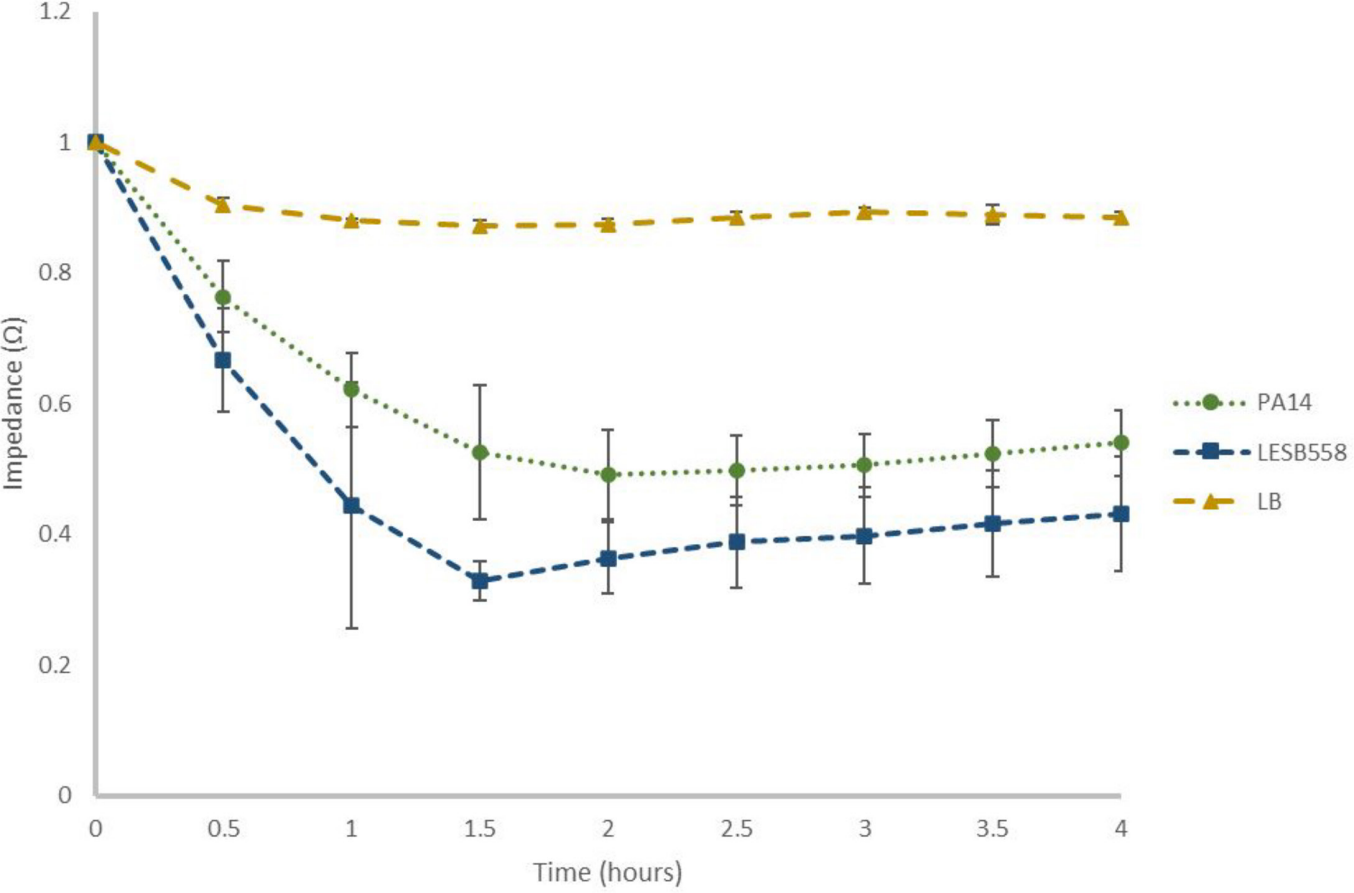
EIS quantification of *P. aeruginosa* biofilms over 4 h. PA14 (green), LESB58 (blue) biofilms and LB media control (yellow) quantified using EIS (10 Hz) over 4 h. Impedance normalized by dividing each data set by their *t*=0 value. Error bars show sd and *n*=3, and * denotes significant difference (*P*<0.05).

As mentioned previously, SWV measurements at −0.35 V quantify the concentration of pyocyanin within the media (correlating to the planktonic growth of *P. aeruginosa*). As such, it was hypothesized that EIS could be employed to quantify biofilm formation, as this instead measured the build-up of cells on the electrode, with a decrease in impedance modulus indicating biofilm formation. As expected, the EIS spectra of PA14 and LESB58 over 4 h showed a decrease in normalized impedance modulus (Ω) from 1 to 0.54 and 1 to 0.43 for PA14 and LESB58, respectively (Fig. 3), indicating that both strains had formed biofilms within the 4 h. Furthermore, there was a significant difference observed in the quantity of biofilm formed by LESB58 after 1.5 h (*P*=0.046, *n*=3), when compared to 0 h, and a significant difference in the quantity of biofilm formed by PA14 after just 1 h (*P*=0.00033, *n*=3) (Fig. 3). Typically, impedance data are fit to a circuit model as a method of normalization [38,39]; however, model fitting was not carried out here, as significant differences between the LB control and both *P. aeruginosa* strains could be observed without this. Instead, the data were normalized, as in a study by Hannah *et al.* [40], by dividing by the corresponding *t*=0 value for each condition. Lastly, there was no significant difference in biofilm between either strain of *P. aeruginosa* after 4 h (*P*=0.076, *n*=3). This is despite other studies indicating that LESB58 is a superior biofilm former, due to its lack of motility [55], and conversely, Fig. S2 showed that PA14 formed more biofilm when quantified with CV; the ability to detect biofilm formation in real time, and within 90 min, for both strains, has strong implications in the field of high-throughput diagnostics, for example, in real-time monitoring of medical implants.

## Discussion

As discussed earlier, there is no standardized method in which biofilms are quantified in the literature, and there is no antibiofilm agent currently available on the US market [1,63]. One of the main inconsistencies within the biofilm-quantification community is variations, which occur within the CV-staining protocol, despite CV being the most used biofilm quantification method [31]. For example, there are additional PBS washes of the biofilm pre-staining [64], post-staining [56], solvent variation [2] and increased concentrations of CV [21,65]. In one of the earlier studies using CV, in 1998, CV was added directly to the media after the biofilms were grown, resulting in planktonic cells also being stained alongside the biofilm [34]. Furthermore, the authors used 1% CV [34], a concentration used in several studies [24,33, 34]. This is in comparison to the 0.1% used in other studies [1,7, 25, 35], including this one. Two of the studies, which used 0.1% CV, looked at reduction in biofilm formation using amino acids [35] and biofilm growth in different media [1], respectively. Both also included photographs alongside quantification of the biofilms with CV to highlight the background staining. These studies also showed clear trends from the CV data, with similar margins of error as here, and larger error at higher absorbance (570 nm) values. This supports the data and the CV quantification protocol presented here, with a lower concentration of CV as a useful method of biofilm quantification, which can inform further assay development with EIS. Lastly, and importantly for the work carried out here, the quantification of biofilm-bound CV is an indirect measurement; the biofilm-bound CV is resolubilized into a solvent, and then, this is measured [7,65]. Here, the aim was to develop a method, which could be used to quantify the biofilm as it was forming, rather than an endpoint method, such as CV. This builds upon work previously presented by Dunphy *et al.*, in which pyocyanin detection was used as a proxy for biofilm formation [38]. Here, biofilm formation was able to be measured directly on the sensor, increasing the translatability of the work into other non-pyocyanin-producing, biofilm-forming pathogens.

Electrochemical measurements, including EIS, have been used to quantify planktonic and biofilm cells previously. As mentioned above, electrochemical data and, in particular, impedance data are fit to an equivalent circuit model, such as Randles [38,39, 66]. However, circuit fitting was not carried out here due to the trends in the data being apparent prior to model fitting. Hannah *et al.* found similarly that, when measuring the planktonic growth of *E. coli* on their gel-electrode system, there was no requirement for model fitting and that changes in bacterial growth could be detected from raw impedance modulus values at 100 kHz [40]. This is highly encouraging, as the removal of part of the workflow allows electrochemical methods to be more accessible and therefore increase the likelihood of them being used in the clinical setting by non-specialists. However, the error associated with increased current values could pose issues of reliability in the clinical setting [67]. Furthermore, increased variation between measurements and controls has previously been attributed to metabolites within the growth media, which are not present in the standards, and increased concentrations of bacteria would hence lead to further variation compared to the standards [38]. Another consideration within a clinical setting is the interference of the EIS measurement by sputum, blood, urine and other body fluids. Recently, EIS has been used to detect a cyclic peptide in environmental water samples [68], medicine within patient blood [69] and human protein within simulated urine [70]. However, if another sample constituent (other bacteria, macromolecules and drugs) is detected at the same potential during SWV or initiates biofouling, then this would give a false positive. Due to the developed method using two electrochemical methods, this makes the measurements more robust against interferences.

This work has demonstrated the benefit of electrochemical methods over conventional methods, such as CV, but also lesser-used methods, such as hyperspectral imaging. A clear limitation of the study presented here is the inability to directly measure planktonic growth, instead inferring from the pyocyanin concentration [38]. However, previous studies have looked at planktonic only [40], or planktonic in one system and biofilm in another [38], rendering the results incomparable. Therefore, this middle ground of using both SWV and EIS must be seen as a compromise that will be overcome. Using EIS to monitor *P. aeruginosa* biofilms has previously shown a decreasing impedance as well, with the authors also monitoring capacitance, which is inversely proportional to impedance [71]. Furthermore, Kretzschmar *et al.* discussed the possibility that carbon electrodes limit the determination of some biofilm properties, due to the increased capacitance associated with the material, subsequently automatically lowering the impedance measurements [71] when compared to other published data on different electrode materials, such as gold, where impedance data could be read at higher frequencies [25]. Lastly, an increased electrical ‘noise’ has been associated with a multiplexer, which also results in higher EIS frequencies being unusable [47], and therefore, studies using single electrodes have been able to monitor higher frequencies [25]. These factors may go some way to explaining why the trends in the EIS spectra here were only seen at lower frequencies (10 Hz).

## Conclusions

From this work, a standardized method for reliable biofilm quantification of two *P. aeruginosa* strains (PA14 and LESB58) has been achieved using SWV and EIS measurements, further showing that the raw impedance modulus reads could give quantifiable measurements during biofilm formation, rather than endpoint measurements only. This is the first time that this has been shown for *P. aeruginosa* biofilms without the need for post-measurement model fitting. This advancement makes * P. aeruginosa* biofilm detection more readily accessible and is a huge step towards *in vivo* quantification. *P. aeruginosa* biofilms have been quantified previously using EIS; however, as mentioned, importantly, the data required circuit fitting [38,72,74]. This increases processing time and makes the technology less accessible for non-specialists [38], thereby limiting its use, something this work circumvents. In fact, to our knowledge, the only other instance of bacterial quantification using the raw impedance values was focussed on *Escherichia coli* [40]. The 2020 study demonstrated the use of impedance measurements for phenotypic antibiotic (streptomycin) susceptibility testing [40] and, as such, demonstrates another exciting potential for real-world applications, for example, real-time antibiotic susceptibility measurements for isolated strains in clinical settings. In future work, it would be of interest to observe how *P. aeruginosa* responds to antibiotic or antibiofilm agents, and if this can be detected with the developed electrochemical system. Biological replicates of this work would help to improve the accuracy. Lastly, these electrochemical techniques have interesting patient-care applications, and it would be highly worthwhile to assay this with medically relevant materials, as a stepping-stone to point-of-care electrochemical sensing that could be used to detect biofilms forming on implanted materials.

## supplementary material

10.1099/acmi.0.000906.v4 Uncited Fig. S1.

## Abbreviations

AMR: antimicrobial resistance
CF: cystic fibrosis
CV: crystal violet
ECM: extracellular matrix
EIS: electrochemical impedance spectroscopy
LB: lysogeny broth
RT: room temperature
SWV: square wave voltammetry

## References

[1] Haney EF, Trimble MJ, Cheng JT, Vallé Q, Hancock REW (2018). Critical assessment of methods to quantify biofilm growth and evaluate antibiofilm activity of host defence peptides. Biomol.

[2] Akinbobola AB, Sherry L, Mckay WG, Ramage G, Williams C (2017). Tolerance of *Pseudomonas aeruginosa* in *in-vitro* biofilms to high-level peracetic acid disinfection. J Hosp Infect.

[3] Anwar H, Costerton JW (1990). Enhanced activity of combination of tobramycin and piperacillin for eradication of sessile biofilm cells of *Pseudomonas aeruginosa*. Antimicrob Agents Chemother.

[4] Moskowitz SM, Foster JM, Emerson J, Burns JL (2004). Clinically feasible biofilm susceptibility assay for isolates of *Pseudomonas aeruginosa* from patients with cystic fibrosis. J Clin Microbiol.

[5] Podos SD, Thanassi JA, Leggio M, Pucci MJ (2012). Bactericidal activity of ACH-702 against nondividing and biofilm *Staphylococci*. Antimicrob Agents Chemother.

[6] Wilson C, Lukowicz R, Merchant S, Valquier-Flynn H, Caballero J (2016). Quantitative and qualitative assessment methods for biofilm growth: a mini-review. Nature Rev Drug Discovery.

[7] Kamer AMA, Abdelaziz AA, Al-Monofy KB, Al-Madboly LA (2023). Antibacterial, antibiofilm, and anti-quorum sensing activities of pyocyanin against methicillin-resistant *Staphylococcus aureus*: *in vitro* and *in vivo* study. BMC Microbiol.

[8] Donlan RM (2001). Biofilm formation: a clinically relevant microbiological process. Clin Infect Dis.

[9] Wang EW, Jung JY, Pashia ME, Nason R, Scholnick S (2005). Otopathogenic *Pseudomonas aeruginosa* strains as competent biofilm formers. Arch Otolaryngol Head Neck Surg.

[10] Fisher RA, Gollan B, Helaine S (2017). Persistent bacterial infections and persister cells. *Nat Rev Microbiol*.

[11] Xu Y, Dhaouadi Y, Stoodley P, Ren D (2020). Sensing the unreachable: challenges and opportunities in biofilm detection. Curr Opin Biotechnol.

[12] Kalia VC, Prakash J, Koul S, Ray S (2017). Simple and rapid method for detecting biofilm forming bacteria. Indian J Microbiol.

[13] Høiby N, Bjarnsholt T, Givskov M, Molin S, Ciofu O (2010). Antibiotic resistance of bacterial biofilms. Int J Antimicrob Agents.

[14] Gilbert-Girard S, Reigada I, Savijoki K, Yli-Kauhaluoma J, Fallarero A (2021). Screening of natural compounds identifies ferutinin as an antibacterial and anti-biofilm compound. *Biofouling*.

[15] Marrie TJ, Costerton JW (1984). Scanning and transmission electron microscopy of in situ bacterial colonization of intravenous and intraarterial catheters. J Clin Microbiol.

[16] Siracuse JJ, Nandivada P, Giles KA, Hamdan AD, Wyers MC (2013). Prosthetic graft infections involving the femoral artery. J Vasc Surg.

[17] Tseng Y-H, Lin C-C, Wong MY, Kao C-C, Lu M-S (2023). *Pseudomonas aeruginosa* infections are associated with infection recurrence in arteriovenous grafts treated with revision. Medicina.

[18] NBIC (2021). National biofilms innovation centre annual report 2021.

[19] Cabral DJ, Wurster JI, Belenky P (2018). Antibiotic persistence as a metabolic adaptation: stress, metabolism, the host, and new directions. Pharm.

[20] Conibear TCR, Collins SL, Webb JS (2009). Role of mutation in *Pseudomonas aeruginosa* biofilm development. PLoS One.

[21] Peeters E, Nelis HJ, Coenye T (2008). Comparison of multiple methods for quantification of microbial biofilms grown in microtiter plates. J Microbiol Methods.

[22] Fletcher M (1977). The effects of culture concentration and age, time, and temperature on bacterial attachment to polystyrene. Can J Microbiol.

[23] Stiefel P, Rosenberg U, Schneider J, Mauerhofer S, Maniura-Weber K (2016). Is biofilm removal properly assessed? comparison of different quantification methods in a 96-well plate system. Appl Microbiol Biotechnol.

[24] Djordjevic D, Wiedmann M, McLandsborough LA (2002). Microtiter plate assay for assessment of *Listeria monocytogenes* biofilm formation. Appl Environ Microbiol.

[25] van Duuren JBJH, Müsken M, Karge B, Tomasch J, Wittmann C (2017). Use of single-frequency impedance spectroscopy to characterize the growth dynamics of biofilm formation in *Pseudomonas aeruginosa*. Sci Rep.

[26] Hancock V, Klemm P (2007). Global gene expression profiling of asymptomatic bacteriuria *Escherichia coli* during biofilm growth in human urine. Infect Immun.

[27] Nilsson M, Chiang W-C, Fazli M, Gjermansen M, Givskov M (2011). Influence of putative exopolysaccharide genes on *Pseudomonas* putida KT2440 biofilm stability. Environ Microbiol.

[28] Guiton PS, Hung CS, Kline KA, Roth R, Kau AL (2009). Contribution of autolysin and Sortase A during *Enterococcus faecalis* DNA-dependent biofilm development. Infect Immun.

[29] Dinda AP, Asnani A, Anjarwati DU (2021). The Activities of Streptomyces W-5A as Antibacterial and Antibiofilm towards Methicillin-resistant *Staphylococcus aureus* 2983.

[30] Milivojevic D, Šumonja N, Medic S, Pavic A, Moric I (2018). Biofilm-forming ability and infection potential of *Pseudomonas aeruginosa* strains isolated from animals and humans. Pathog Dis.

[31] Summer K, Browne J, Hollanders M, Benkendorff K (2022). Out of control: The need for standardised solvent approaches and data reporting in antibiofilm assays incorporating dimethyl-sulfoxide (DMSO). *Biofilm*.

[32] Mangzira Kemung H, Tan LT-H, Chan K-G, Ser H-L, Law JW-F (2020). *Streptomyces* sp. Strain MUSC 125 from mangrove soil in Malaysia with Anti-MRSA, anti-biofilm and antioxidant activities. Molecules.

[33] Woodward MJ, Sojka M, Sprigings KA, Humphrey TJ (2000). The role of SEF14 and SEF17 fimbriae in the adherence of *Salmonella* enterica serotype *Enteritidis* to inanimate surfaces. J Med Microbiol.

[34] O’Toole GA, Kolter R (1998). Initiation of biofilm formation in *Pseudomonas fluorescens* WCS365 proceeds via multiple, convergent signalling pathways: a genetic analysis. Mol Microbiol.

[35] Kao WTK, Frye M, Gagnon P, Vogel JP, Chole R (2017). D-amino acids do not inhibit *Pseudomonas aeruginosa* biofilm formation. Laryngoscope Investig Otolaryngol.

[36] Panda PS, Chaudhary U, Dube SK (2016). Comparison of four different methods for detection of biofilm formation by uropathogens. Indian J Pathol Microbiol.

[37] Domingo-Roca R, Lasserre P, Riordan L, Macdonald AR, Dobrea A (2023). Rapid assessment of antibiotic susceptibility using a fully 3D-printed impedance-based biosensor. Biosens Bioelectron.

[38] Dunphy RD, Lasserre P, Riordan L, Duncan KR, McCormick C (2022). Combining hyperspectral imaging and electrochemical sensing for detection of *Pseudomonas aeruginosa* through pyocyanin production. Sens Diagn.

[39] Hannah S, Addington E, Alcorn D, Shu W, Hoskisson PA (2019). Rapid antibiotic susceptibility testing using low-cost, commercially available screen-printed electrodes. Biosens Bioelectron.

[40] Hannah S, Dobrea A, Lasserre P, Blair EO, Alcorn D (2020). Development of a rapid, antimicrobial susceptibility test for *E. coli* based on low-cost, screen-printed electrodes. Biosensors.

[41] Macdonald JR (1992). Impedance spectroscopy. Ann Biomed Eng.

[42] Ramírez N, Regueiro A, Arias O, Contreras R (2009). Electrochemical impedance spectroscopy: an effective tool for a fast microbiological diagnosis. Biotecnol Apl.

[43] Alatraktchi FA, Andersen SB, Johansen HK, Molin S, Svendsen WE (2016). Fast selective detection of pyocyanin using cyclic voltammetry. Sensors.

[44] Bellin DL, Sakhtah H, Zhang Y, Price-Whelan A, Dietrich LEP (2016). Electrochemical camera chip for simultaneous imaging of multiple metabolites in biofilms. Nat Commun.

[45] Oziat J, Cohu T, Elsen S, Gougis M, Malliaras GG (2021). Electrochemical detection of redox molecules secreted by *Pseudomonas aeruginosa* - Part 1: electrochemical signatures of different strains. Bioelectrochem.

[46] Kim T, Kang J, Lee JH, Yoon J (2011). Influence of attached bacteria and biofilm on double-layer capacitance during biofilm monitoring by electrochemical impedance spectroscopy. Water Research.

[47] Barreiro M, Sánchez P, Vera J, Viera M, Morales I (2022). Multiplexing error and noise reduction in electrical impedance tomography imaging. Front Electron.

[48] Pellé J, Longo M, Le Poul N, Hellio C, Rioual S (2023). Electrochemical monitoring of the *Pseudomonas aeruginosa* growth and the formation of a biofilm in TSB media. Bioelectrochem.

[49] Dawadi P, Khadka C, Shyaula M, Syangtan G, Joshi TP (2022). Prevalence of metallo-β-lactamases as a correlate of multidrug resistance among clinical *Pseudomonas aeruginosa* isolates in Nepal. Sci Total Environ.

[50] Ochoa SA, López-Montiel F, Escalona G, Cruz-Córdova A, Dávila LB (2013). Características patogénicas de cepas de *pseudomonas aeruginosa* resistentes a carbapenémicos, asociadas con la formación de biopelículas. Bol Med Hosp Infant Mex.

[51] Stover CK, Pham XQ, Erwin AL, Mizoguchi SD, Warrener P (2000). Complete genome sequence of *Pseudomonas aeruginosa* PAO1, an opportunistic pathogen. Nature.

[52] de Bentzmann S, Plésiat P (2011). The *Pseudomonas aeruginosa* opportunistic pathogen and human infections. Environ Microbiol.

[53] Moore MP, Lamont IL, Williams D, Paterson S, Kukavica-Ibrulj I (2021). Transmission, adaptation and geographical spread of the *Pseudomonas aeruginosa* Liverpool epidemic strain. Microb Genom.

[54] Lee DG, Urbach JM, Wu G, Liberati NT, Feinbaum RL (2006). Genomic analysis reveals that *Pseudomonas aeruginosa* virulence is combinatorial. Genome Biol.

[55] Kukavica-Ibrulj I, Bragonzi A, Paroni M, Winstanley C, Sanschagrin F (2008). In vivo growth of *Pseudomonas aeruginosa* strains PAO1 and PA14 and the hypervirulent strain LESB58 in a rat model of chronic lung infection. J Bacteriol.

[56] Mikkelsen H, McMullan R, Filloux A (2011). The *Pseudomonas aeruginosa* reference strain PA14 displays increased virulence due to a mutation in ladS. PLoS One.

[57] Martin K, Baddal B, Mustafa N, Perry C, Underwood A (2013). Clusters of genetically similar isolates of *Pseudomonas aeruginosa* from multiple hospitals in the UK. J Med Microbiol.

[58] Cheng K, Smyth RL, Govan JR, Doherty C, Winstanley C (1996). Spread of beta-lactam-resistant *Pseudomonas aeruginosa* in a cystic fibrosis clinic. Lancet.

[59] Winstanley C, Langille MGI, Fothergill JL, Kukavica-Ibrulj I, Paradis-Bleau C (2009). Newly introduced genomic prophage islands are critical determinants of in vivo competitiveness in the liverpool epidemic strain of *Pseudomonas aeruginosa*. Genome Res.

[60] Passador L, Cook JM, Gambello MJ, Rust L, Iglewski BH (1993). Expression of *Pseudomonas aeruginosa* virulence genes requires cell-to-cell communication. Science.

[61] Webster TA, Sismaet HJ, Conte JL, Chan IJ, Goluch ED (2014). Electrochemical detection of *Pseudomonas aeruginosa* in human fluid samples via pyocyanin. Biosens Bioelectron.

[62] Meier NR, Sutter TM, Jacobsen M, Ottenhoff THM, Vogt JE (2020). Machine learning algorithms evaluate immune response to Novel *Mycobacterium tuberculosis* antigens for diagnosis of tuberculosis. Front Cell Infect Microbiol.

[63] Condren AR, Kahl LJ, Boelter G, Kritikos G, Banzhaf M (2020). Biofilm Inhibitor taurolithocholic acid alters colony morphology, specialized metabolism, and virulence of *Pseudomonas aeruginosa*. ACS Infect Dis.

[64] Topa SH, Palombo EA, Kingshott P, Blackall LL (2020). Activity of cinnamaldehyde on quorum sensing and biofilm susceptibility to antibiotics in *Pseudomonas aeruginosa*. Microorganisms.

[65] Yahya MFZR, Alias Z, Karsani SA (2018). Antibiofilm activity and mode of action of DMSO alone and its combination with afatinib against Gram-negative pathogens. Folia Microbiol.

[66] Russell C, Ward AC, Vezza V, Hoskisson P, Alcorn D (2019). Development of a needle shaped microelectrode for electrochemical detection of the sepsis biomarker interleukin-6 (IL-6) in real time. Biosens Bioelectron.

[67] Cornish-Bowden A (1982). Fundamentals of Enzyme Kinetics, 4th ed.

[68] Mandal AK, Pal T, Kumar S, Mukherji S, Mukherji S (2024). A portable EIS-based biosensor for the detection of microcystin-LR residues in environmental water bodies and simulated body fluids. Analyst.

[69] Ozkan E, Ozcelikay G, Gök Topak ED, Nemutlu E, Ozkan SA (2023). Molecularly imprinted electrochemical sensor for the selective and sensitive determination of octreotide in cancer patient plasma sample. Talanta.

[70] Alharthi SD, Kanniyappan H, Prithweeraj S, Bijukumar D, Mathew MT (2023). Proteomic-based electrochemical non-invasive biosensor for early breast cancer diagnosis. Int J Biol Macromol.

[71] Kretzschmar J, Harnisch F (2021). Electrochemical impedance spectroscopy on biofilm electrodes – conclusive or euphonious?. Curr Opin Eectrochemistry.

[72] Chabowski K, Junka AF, Szymczyk P, Piasecki T, Sierakowski A (2015). The application of impedance microsensors for real-time analysis of *Pseudomonas aeruginosa* biofilm formation. Pol J Microbiol.

[73] Ward AC, Connolly P, Tucker NP (2014). *Pseudomonas aeruginosa* can be detected in a polymicrobial competition model using impedance spectroscopy with a novel biosensor. PLoS One.

[74] Hannah AJ, Ward AC, Connolly P (2021). Rapidly detected common wound pathogens via easy-to-use electrochemical sensors. J Biomed Eng Biosci.

