# Supplementary material for: Quantification of Pseudomonas aeruginosa biofilms using electrochemical methods
Source: Access Microbiol. 2025 Feb 14;7(2):000906.v4. doi: 10.1099/acmi.0.000906.v4 (PMC11829079; doi:10.1099/acmi.0.000906.v4)
Supplement: Uncited Fig. S1. [file acmi-7-00906-s001.pdf]

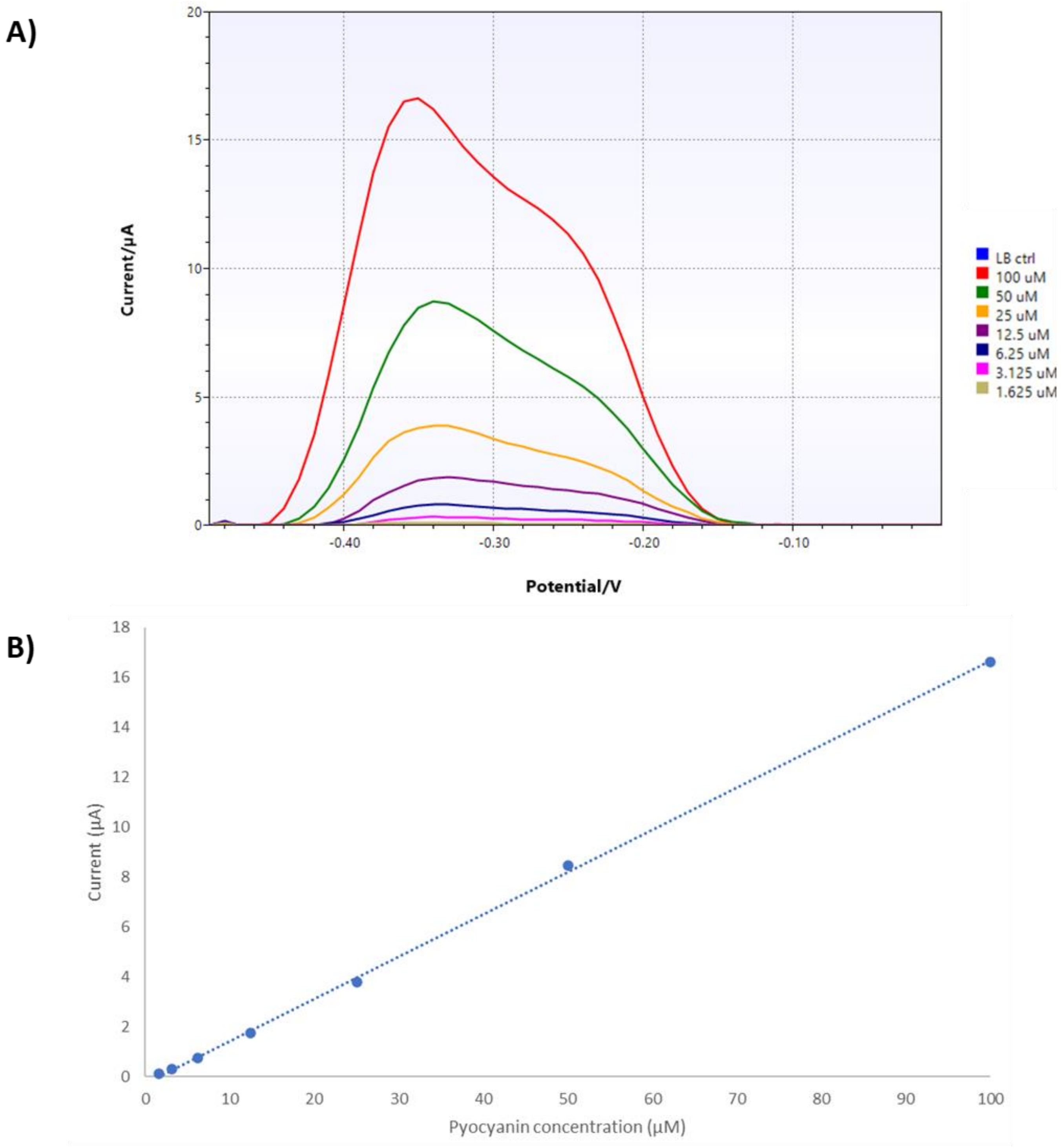

Supplementary Figure 1: SWV Pyocyanin concentration curve. Pyocyanin serial diluted in LB (1.56 – 100 µM) and **A)** measured across a range of potential differences (V). **B)** Corresponding pyocyanin concentration curve showing increasing current output (µA) with increasing pyocyanin concentrations at -0.35 V, r^2=99.9%.

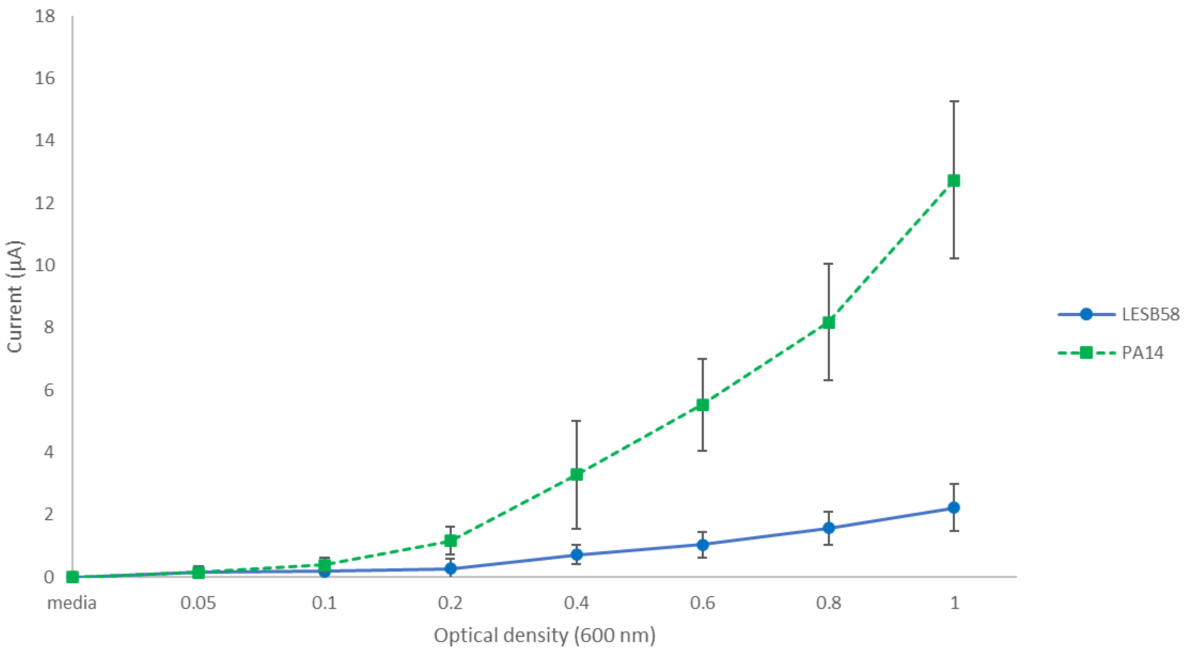

Supplementary Figure 2: SWV quantification of *P. aeruginosa* (PA14 and LESB58) with increasing seeding densities (OD600 0.05 – 1). Current (µA) measured at -0.35 V over a four-hour incubation, pre-normalised four-hour time points shown (error bars show standard deviation, n=3, r^2=91%).
